# Designed ankyrin repeat proteins are effective targeting elements for chimeric antigen receptors

**DOI:** 10.1186/s40425-015-0099-4

**Published:** 2015-12-15

**Authors:** Joanne A. Hammill, Heather VanSeggelen, Christopher W. Helsen, Galina F. Denisova, Carole Evelegh, Daniela G. M. Tantalo, Jennifer D. Bassett, Jonathan L. Bramson

**Affiliations:** Department Pathology and Molecular Medicine, McMaster Immunology Research Centre, McMaster University, Hamilton, ON Canada

**Keywords:** Cancer, Immunotherapy, Chimeric antigen receptor, CAR, Designed ankyrin repeat protein, DARPin

## Abstract

**Background:**

Adoptive cell transfer of tumor-specific T lymphocytes (T cells) is proving to be an effective strategy for treating established tumors in cancer patients. One method of generating these cells is accomplished through engineering bulk T cell populations to express chimeric antigen receptors (CARs), which are specific for tumor antigens. Traditionally, these CARs are targeted against tumor antigens using single-chain antibodies (scFv). Here we describe the use of a designed ankyrin repeat protein (DARPin) as the tumor-antigen targeting domain.

**Methods:**

We prepared second generation anti-HER2 CARs that were targeted to the tumor antigen by either a DARPin or scFv. The CARs were engineered into human and murine T cells. We then compared the ability of CARs to trigger cytokine production, degranulation and cytotoxicity.

**Results:**

The DARPin CARs displayed reduced surface expression relative to scFv CARs in murine cells but both CARs were expressed equally well on human T cells, suggesting that there may be a processing issue with the murine variants. In both the murine and human systems, the DARPin CARs were found to be highly functional, triggering cytokine and cytotoxic responses that were similar to those triggered by the scFv CARs.

**Conclusions:**

These findings demonstrate the utility of DARPins as CAR-targeting agents and open up an avenue for the generation of CARs with novel antigen binding attributes.

## Background

Cancer immunotherapy aims to treat tumors by engaging the patient’s immune system. One form of immunotherapy, called adoptive cell transfer, infuses cancer patients with a bolus of tumor-specific T lymphocytes (T cells), and is proving to be an effective treatment for a variety of malignancies [1–3]. In adoptive cell transfer, T cells isolated from a tumor-bearing patient are grown to large numbers *ex vivo* and are administered back into the patient to induce a robust anti-tumor immune response. Tumor specificity can be achieved by either i) isolating naturally occurring tumor-specific T cells from the patient, or ii) engineering bulk T cells from the peripheral blood to express tumor-specific receptors on their surface. Naturally-occurring tumor-specific T cells are rare and expanding them from a cancer patient is typically a laborious procedure. In contrast, it is becoming relatively easy to engineer readily-available peripheral T cells with tumor-specific receptors through genetic manipulation. Techniques have been developed for this engineering process which are clinically-viable and multiple clinical trials have demonstrated feasibility and efficacy of genetically-engineered T cells for the treatment of cancer [1, 3–9].

Chimeric antigen receptors (CARs), recombinant proteins designed for expression on the surface of T cells, offer one way to engineer T cells with anti-tumor functionality. CARs are composed of an extracellular antigen recognition domain linked to intracellular signaling domains derived from the T cell receptor and co-receptors (including combinations of the signaling regions of CD3ζ, CD28, and/or 4-1BB, for example) such that the T cells become activated following binding of tumor antigen by the CAR. Depending upon the nature of the intracellular signaling domains, this activation event can lead to cytokine production, cytotoxic attack of the tumor, and proliferation of the T cells.

Most CARs developed to date, including those specific for the tumor associated antigens human epidermal growth factor receptor 2 (HER2) [4, 10] and CD19 [3, 7, 8], utilize a single-chain variable fragment (scFv), derived from an antibody, to enable antigen recognition. However, scFvs do not represent the sole or, necessarily, the optimal option for antigen targeting of CARs.

Ankyrin repeats (ARs), one of the most common protein motifs found in nature, are 33 amino acid long sequences composed of a β-turn followed by two anti-parallel α-helices and a loop [11, 12]. Various numbers of these individual ARs stack together to form ankyrin repeat proteins which function as protein binders [11, 13]. Recognizing the potential of these natural ankyrin repeat proteins as alternative target-binding domains, libraries of artificial stacked ARs, called designed ankyrin repeat proteins (DARPins) were developed to allow for the generation of repeat protein binders against a defined target of interest [14, 15]. Each DARPin in these libraries typically consists of between 2 and 6 repeating units; 2–4 repeats containing both fixed (framework sites required for correct AR folding) and variable (randomized sites leading to a diversity of target-binding capacity within the library) amino acid positions sandwiched between non-variable N-terminal and C-terminal capping repeats (essential for correct DARPin folding) [16, 17]. Expression of these genetic DARPin libraries using ribosome or phage display systems allows for the selection of DARPins with the capacity to bind a defined target of interest as well as refine binding affinity for that target [18].

DARPins offer a number of features which make them attractive for use in the CAR field: 1) they are more compact than scFvs and, thus, take up less space in the genetic transfer vectors typically used for engineering T cells (ex. retrovirus and lentivirus), 2) they are very thermodynamically stable, and 3) they do not require pairing of separate binding domains (e.g. V_H_ and V_L_ of the scFv), allowing the facile linkage of multiple DARPins, with different specificities, which could be used to create a multi-specific CAR.

We tested the utility of DARPins to target CARs using a DARPin specific for the tumor associated antigen HER2 [19]. As a gold standard, we employed an scFv against HER2 [20]. Both targeting elements were incorporated into murine and human CAR scaffolds to rigorously test the suitability of DARPins. Our results demonstrated that targeting CARs with DARPins is as efficacious as targeting CARs with scFvs and supports the further investigation of DARPin CARs.

## Results and discussion

### Expression of DARPin28z on murine and human T lymphocytes

**Fig. 1:**
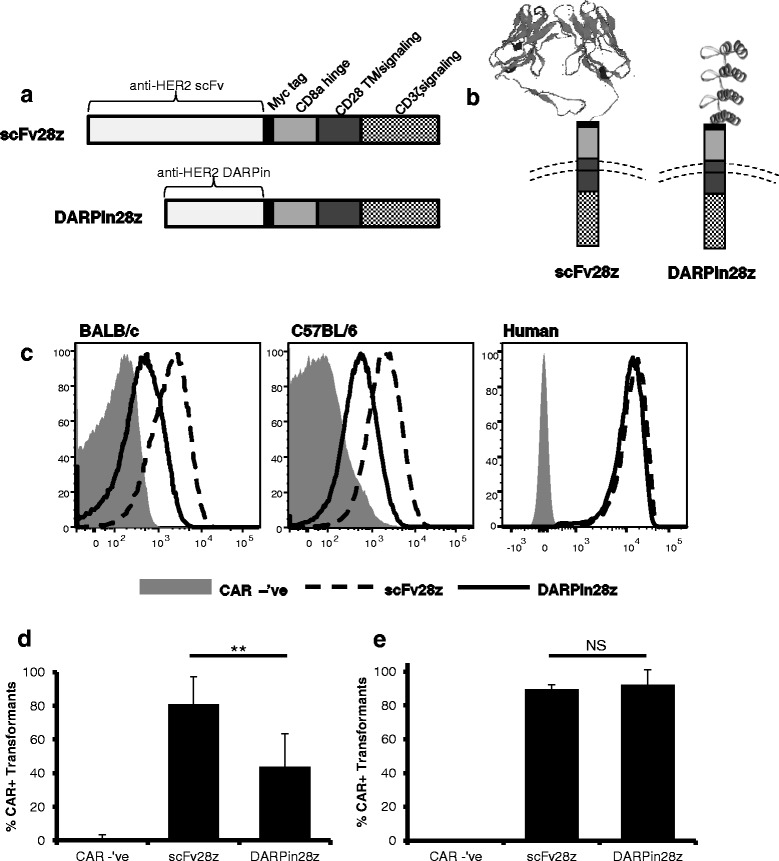
Expression of scFv28z and DARPin28z on the surface of murine and human T lymphocytes. **a** Schematic representation of CAR structures. Each construct was composed of an antigen recognition domain (either scFv or DARPin), specific for HER2, fused to a myc tag, CD8α hinge, CD28 transmembrane and signaling domains, and the signaling portion of CD3ζ. Identical constructs were generated for expression in murine or human T cells, using species specific sequences for CAR components. **b** Schematic showing orientation of CAR structures in relation to the T cell surface. Ribbon diagrams for the scFv and DARPin domains illustrate differences in tertiary structure and size. **c**-**e** CAR expression on the surface of murine (BALB/c or C57BL/6) or human T cells was analyzed by flow cytometry. All plots show virally transduced CD8^+^ lymphocytes (CAR-expressing viruses also expressed transduction markers; Thy1.1 for murine constructs and NGFR for human constructs). CAR negative cells were used as controls; T cells were transduced with constructs expressing transduction markers in the absence of a CAR. CAR expression was measured by staining with an α-myc tag antibody, or a HER2Fc fusion protein, followed by a secondary detection antibody. **c.** Representative plots are shown. **d**-**e** Level of CAR expression by transduced cells; calculated for murine ((**d**) % CAR^+^/% Thy1.1^+^) and human ((**e**) % CAR^+^/NGFR^+^) CAR-T cell cultures. Data representative of multiple experiments ((**d**) murine: CAR –’ve *n* = 3, scFv28z *n* = 6, DARPin28z *n* = 6) ((**e**) human: two donors, *n* = 3 each for CAR –’ve, scFv28z, DARPin28z). Error bars = standard deviation (SD). * = *p* < 0.05 ** = *p* < 0.005 *** = *p* < 0.001

To generate a chimeric antigen receptor which uses a DARPin for its antigen recognition domain, we exchanged the scFv domain of a second generation CAR with specificity against HER2 [20] (herein referred to as scFv28z) with a HER2-specific DARPin [19] (herein referred to as DARPin28z) (Fig. 1a, b). We created both a murine and a human version of both CARs to allow for testing of the DARPin antigen-binding domain in T cells from both species. We noted that the DARPin28z CAR displayed reduced surface expression on T cells from both C57BL/6 and BALB/c mice relative to the scFv28z CAR (Fig. 1c, d). While we do not know the reason for the reduced surface expression, the effect appeared to be related to the murine system because the human DARPin28z receptors were expressed at high levels on T cells from two different donors and displayed surface expression levels equivalent to the scFv28z CAR in the human T cells (Fig. 1c, e). Together these data indicate that DARPin28z is successfully expressed on the surface of both murine and human T lymphocytes.

### DARPin28z induces cytokine production by CAR-T cells upon antigen binding

**Fig. 2:**
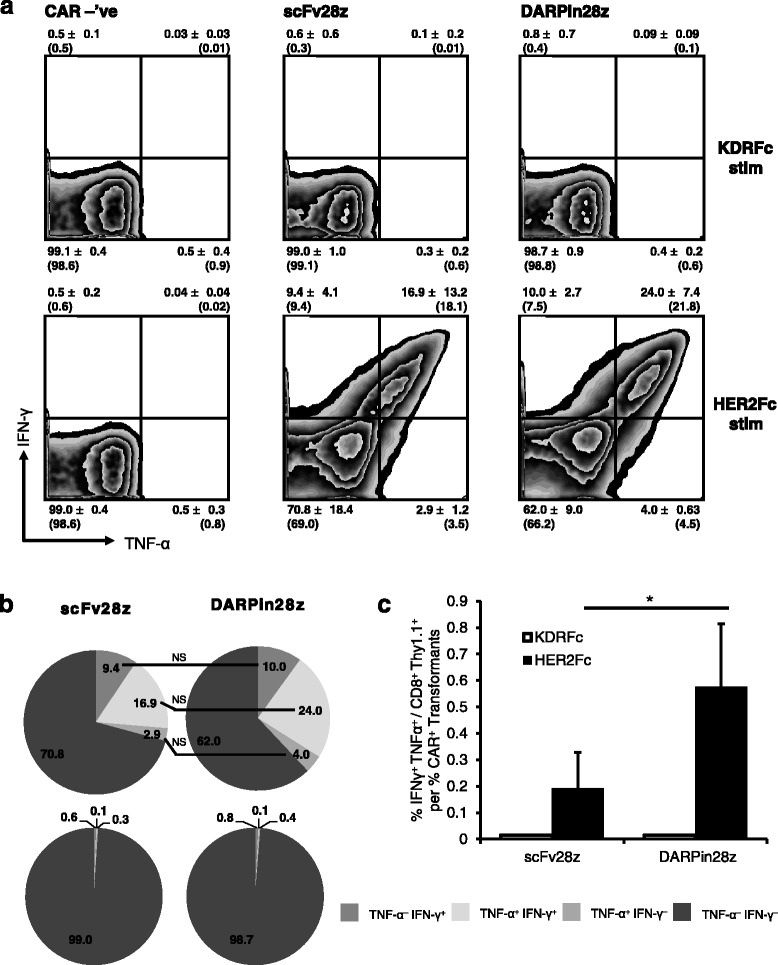
DARPin28z induces murine CAR-T cell cytokine production upon HER2 stimulation. **a** 10^6^ scFv28z, DARPin28z, or CAR –’ve transduced murine T cells were stimulated with HER2 (HER2Fc fusion protein) or an unrelated target (KDRFc fusion protein) for four hours at 37 °C in a 96-well plate. Production of IFN-γ and TNF-α was measured by intracellular cytokine staining (ICS) and subsequent flow cytometry. Data from CD8^+^ Thy1.1^+^ T cells is presented as mean of *n* = 3 experiments (CAR –’ve) and *n* = 5 experiments (scFv28z and DARPin28z) ± SD. Bracketed numbers are quantitative of the representative plots shown. **b** Visual comparison of cytokine production data from a. **c** Cytokine expression data expressed relative to CAR-positivity of transduced cells where CAR^+^ transformant = % CAR^+^/% Thy1.1^+^. Error bars = SD. * = *p* < 0.05 ** = *p* < 0.005 *** = *p* < 0.001

**Fig. 3:**
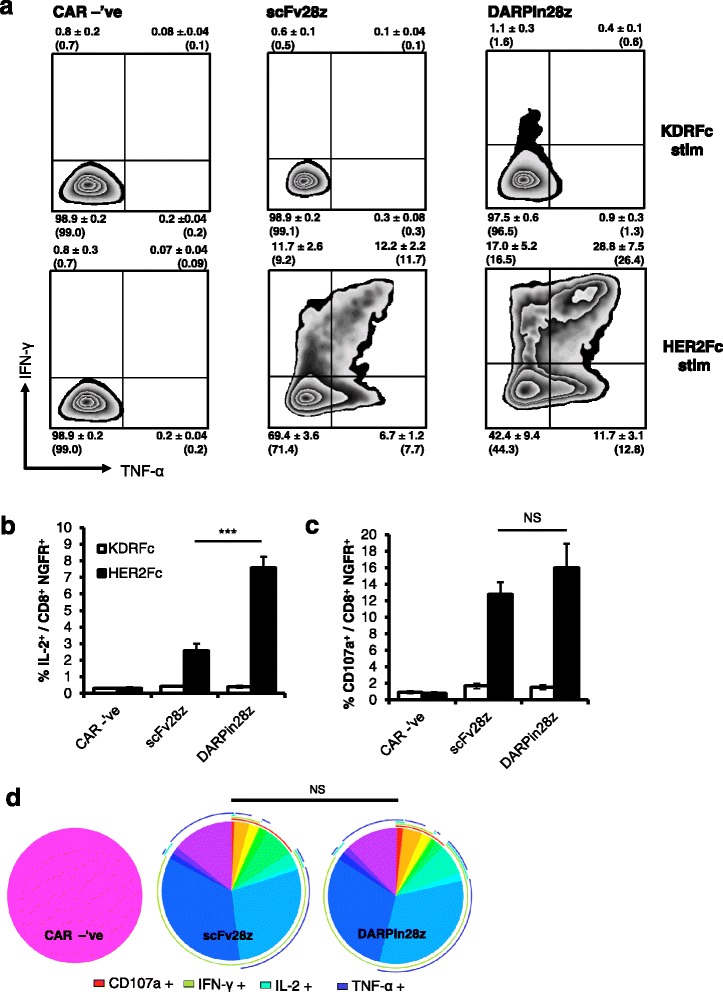
DARPin28z induces human CAR-T cell cytokine production upon HER2 stimulation. 10^6^ scFv28z, DARPin28z, or CAR –’ve transduced human T cells were stimulated with HER2 (HER2Fc fusion protein) or an unrelated target (KDRFc fusion protein) for four hours at 37 °C in a 96-well plate. **a** Production of IFN-γ and TNF-α was measured by ICS and subsequent flow cytometry. Data from CD8^+^ NGFR^+^ T cells is presented as mean ± SD. Bracketed numbers are quantitative of the representative plots shown. **b** Production of IL-2 by CD8^+^ NGFR^+^ T cells as measured by ICS. **c** Production of CD107a by CD8^+^ NGFR^+^ T cells as measured by ICS. **d** Pie graphs capturing the distribution of single and multi-functional CAR-T cells as produced with SPICE software. Pie arcs indicate functional populations represented by pie wedges. Error bars = SD. * = *p* < 0.05 ** = *p* < 0.005 *** = *p* < 0.001. All data from *n* = 3 experiments repeated with T cells from two donors

To test the functionality of the DARPin28z CARs, we stimulated the engineered murine and human T cells with recombinant HER2 or an unrelated control protein (recombinant kinase insert domain receptor (KDR), also known as vascular endothelial growth factor receptor 2 (VEGFR-2)) and measured the production of cytokines using flow cytometry. Murine T lymphocytes expressing the scFv28z CAR produced both IFN-γ and TNF-α upon stimulation with HER2, as did the same cells expressing the DARPin28z CAR (Fig. 2a). Both scFv28z and DARPin28z were able to trigger IFN-γ and TNF-α production in a similar proportion of retrovirally engineered T cells (those expressing the transduction marker Thy1.1) (Fig. 2b). However, the level of CAR expression by retrovirally transduced (Thy1.1 positive) cells varied significantly between scFv28z and DARPin 28z CAR-T cells (Fig. 1d), and when cytokine production data was normalized for CAR-expression, DARPin28z was more effective at inducing IFN-γ and TNF-α double-producing T cells on a per-CAR-T cell basis (Fig. 2c). Indeed, similar results were observed for human T cells; DARPin28z was able to trigger production of both IFN-γ and TNF-α by human T cells (Fig. 3a). In fact, in human T lymphocytes, DARPin28z proved more efficient than scFv28z at inducing cells producing IFN-γ and TNF-α among NGFR-positive cells (lentivirally engineered T cells); IFN-γ^+^ TNF-α^–^: 11.7 ± 2.6 vs 17.0 ± 5.2 (*p* < 0.05), IFN-γ^+^ TNF-α^+^: 12.2 ± 2.2 vs 28.8 ± 7.5 (*p* < 0.001), IFN-γ^–^ TNF-α^+^: 6.7 ± 1.2 vs 11.7 ± 3.1 (*p* < 0.05). Furthermore, DARPin28z was also more efficient at inducing production of IL-2 by human CAR-T cell cultures (Fig. 3b), although showed no enhanced capacity to induce degranulation of HER2 stimulated CAR-T cells, as determined by CD107a release (Fig. 3c). Since the T cell populations are comprised of a constellation of cells with distinct functional phenotypes, we also employed SPICE analysis to determine whether the two CARs selectively activated a particular subpopulation of cells, but this analysis revealed no preferential activation of a particular subpopulation by either CAR (Fig. 3d).

Observed differences in the magnitudes of response may be explained by variances in target binding by the anti-HER2 DARPin vs anti-HER2 scFv. For example, the anti-HER2 DARPin utilized here has an affinity for HER2 of 0.070nM [21] while the scFv has an affinity of 7.2nM [22]. In addition, the scFv used to generate our scFv28z CAR (FRP5 [20, 22]) binds to the distal-most extracellular loops of HER2 [23], whereas the anti-HER2 DARPin used to generate our DARPin28z CAR binds HER2 proximal to the cell membrane [24], which may influence epitope availability. While these differences negate any direct comparisons between DARPin28z and scFv28z efficacy, the above data reveals that the DARPin28z CAR demonstrates an equivalent capacity to activate T cell effector functions, specifically cytokine production, as the scFv28z CAR.

### DARPin28z induces CAR-T cell cytotoxicity against HER2-positive tumor cells

**Fig. 4:**
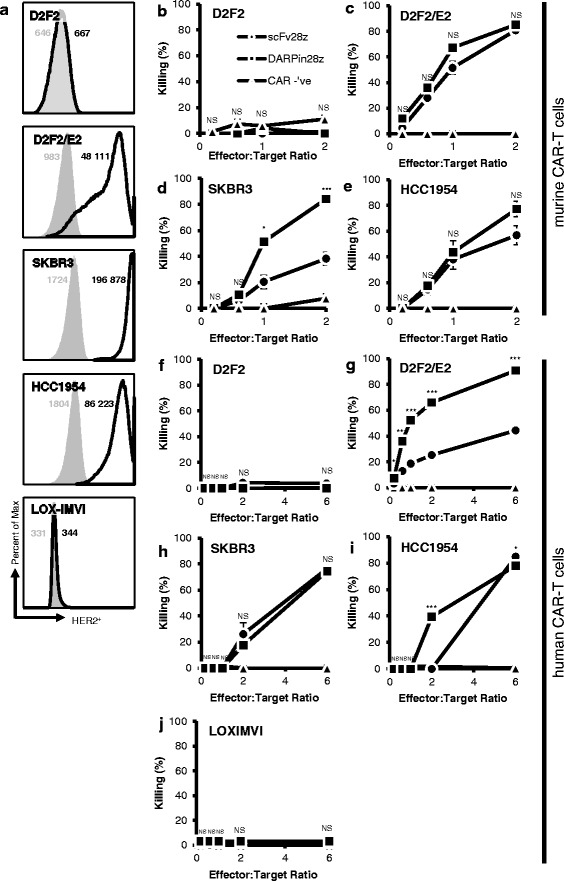
DARPin28z murine and human CAR-T cells are capable of killing HER2^+^ tumor cells. **a** Expression of HER2 on a panel of five tumor cell lines was verified by flow cytometry using a primary anti-HER2 antibody followed by a secondary detection antibody (thick lines). Shaded histograms indicate secondary only controls and numbers indicate mean fluorescence intensity. **b**-**e** Transduced murine T cells (effectors) were incubated together with tumor cells (targets) at various E:T ratios for 6 h in a 96-well plate. Each E:T was tested in triplicate. Error bars = standard error (SE). **f**-**j** Human CAR-T cells were incubated together with tumor cells at various E:T ratios for 6 h. Each E:T was tested in triplicate, data shown is the mean from *n* = 3 experiments repeated with T cells from two donors. Error bars = SE. Statistics compare scFv28z vs DARPin28z (* = *p* < 0.05 ** = *p* < 0.005 *** = *p* < 0.001)

To test the capacity of our DARPin28z CAR-T cells to induce cytotoxicity against HER2-positive tumor cell targets, engineered murine and human CAR-T cells were incubated with a variety of HER2-positive and HER2-negative tumor cell lines; viability of the cell lines was measured 6 h later. HER2-positive cell lines included murine D2F2/E2, a murine breast carcinoma engineered to express human HER2, as well as SKBR3 and HCC1954, human breast carcinomas which naturally overexpress HER2. HER2-negative cell lines included D2F2, a murine breast carcinoma (the parental line of D2F2/E2), and LOX-IMVI, a human melanoma cell line. HER2 expression status on tumor cells was verified via flow cytometry (Fig. 4a). Murine DARPin28z CAR-T cells showed minimal killing of HER2-negative tumor cells (Fig. 4b) but were able to kill HER2-positive tumor cells at effector(E):target(T) ratios of as low as 0.6 T cells per tumor cell (D2F2/E2 and HCC1954, Fig. 4c, e) and 1 T cell per tumor cell (SKBR3, Fig. 4d). Levels of tumor cell killing were similar between scFv28z and DARPin28z CAR-T cells, with the exception of SKBR3 tumor cells, against which DARPin28z CAR-T cells showed increased cytotoxicity compared to scFv28z (Fig. 4d). Human DARPin28z CAR-T cells behaved similarly; HER2-negative tumor cells showed minimal cell death after incubation with CAR-T cells at all E:T ratios tested (Fig. 4f, j) while HER2-positive tumor cells showed evidence of cytotoxicity starting at E:T ratios of 0.1 CAR-T cells per tumor cell (D2F2/E2, Fig. 4g) and 2 CAR-T cells per tumor cell (SKBR3, HCC1954, Fig. 4h, i). Human DARPin28z CAR-T cells were equally as cytotoxic as their scFv28z counterparts against SKBR3 targets (Fig. 4f), but showed superior induction of cytotoxicity against D2F2/E2 targets at all E:T ratios tested (Fig. 4g) and HCC1954 targets at a 2:1 E:T ratio (Fig. 4i). As such, we can conclude that the DARPin28z CAR is capable of activating T cells to induce cytotoxicity against a HER2-positive tumor target while sparing target-negative cells. These data, similar to the results generated following stimulation with recombinant HER-2, confirm that the DARPin28z receptors demonstrate biological activity that is similar to the scFv28z receptor.

## Conclusions

These experiments position DARPin-targeted CARs as a suitable alternative to their scFv-targeted counterparts and strongly support the further investigation of DARPins for use targeting CARs; by all measures investigated, DARPin28z performed similarly to scFv28z as a mechanism for targeting T cells against a HER2-positive target.

In our opinion, DARPins offer a number of attractive features as an antigen-targeting domain. First, DARPins are smaller than scFvs; our anti-HER2 scFv is 739 bp [20] compared to 408 bp for the anti-HER2 DARPin [19]. Since lentivirus titers are often inversely correlated with the size of the lentiviral insert [25, 26], the ability to conserve coding sequence in the lentivirus insert is a desirable feature afforded by using DARPins. Second, it has been argued that DARPins are poorly immunogenic [16, 17], a useful property for the safety and longevity of DARPin-based CAR-T cell therapies. Finally, ARs are amenable to stacking; the number of ARs stacked consecutively in a single protein ranges from one to 33 [11]. As such, we postulate that several DARPin molecules, each with unique antigen-binding properties, could be stacked consecutively to generate a single CAR with the capacity to identify multiple tumor-targets.

## Methods

### Cell lines

Parental D2F2 and human HER2 expressing D2F2/E2 murine mammary tumor cell lines (provided by Dr. Wei-Zen Wei, Barbara Ann Karmanos Cancer Institute, Detroit, MI) were cultured in hi-glucose DMEM supplemented with 5 % FBS (Gibco; Life Technologies, Grand Island, NY), 5 % cosmic calf serum (Fisher Scientific, Waltham, MA), 2.4 mM L-glutamine (BioShop Canada Inc., Burlington, ON), 0.12 mM non-essential amino acids (Gibco), 120U/mL penicillin (Gibco), 120 μg/mL streptomycin (Gibco), 55 μM β-mercaptoethanol (Gibco), and 0.6 mM sodium pyruvate (Sigma-Aldrich Canada Co., Oakville, ON). D2F2/E2 media contained 800 μg/mL geneticin (Gibco). The human tumor cell lines SKBR3, HCC1954, and LOX-IMVI (provided by Dr. Karen Mossman, McMaster University, Hamilton, ON) were cultured in RPMI 1640 supplemented with 10 % FBS, 2 mM L-glutamine, 10 mM HEPES (Roche Diagnostics, Laval, QC), 100U/mL penicillin, 100 μg/mL streptomycin, and 55 μM β-mercaptoethanol. All cell lines were grown at 5 % CO_2_, 95 % air, and 37 °C.

### Generation of CAR retroviruses

A murine anti-HER2 scFv CAR (murine scFv28z) was synthesized at Genscript using the FRP5 scFv sequence (kindly provided by Dr. Phillip K Darcy (University of Melbourne, Parkville, Victoria, Australia) [27]; the scFv [20], was linked to a marker epitope from human *c-myc*, the membrane proximal hinge region of murine CD8, the transmembrane and cytoplasmic regions of murine CD28, and the cytoplasmic region of murine CD3ζ (Table 1). The murine scFv28z was cloned into the retroviral vector pRV2011 oFL (used for the generation of CAR –‘ve transduced murine T cells) [28] (kindly provided by Dr. Brian Rabinovich, MD Anderson, Houston, TX, USA) which also encodes that Thy1.1 marker gene. To generate DARPin28z, the FRP5 sequence in murine scFv28z was replaced with the sequence for the G3 anti-HER2 DARPin [19] [RCSB Protein Data Bank: 2jab]. The G3 DARPin sequence was synthesized at Genscript (Piscataway, NJ, USA). Retroviral supernatants were generated by transient transfection of a packaging cell line with pRV2011 CAR vectors. CAR retroviral vectors (10 μg) and the packaging plasmid pCL-Eco (10 μg) were co-transfected into PLAT-E cells using Lipofectamine 2000 (Invitrogen; Life Technologies). Retrovirus containing supernatants were collected 48 h later and concentrated 40-fold using an Amicon Ultra 100 K centrifugal filter (Millipore (Canada) Ltd., Etobicoke, ON); this process was repeated at 72 h.

**Table 1:** Amino acid sequences of CAR domains. The ten n-terminal and ten c-terminal amino acids which flank the protein regions utilized as CAR domains (with the exception of c-myc for which the sequence is listed in its entirety)

Murine CAR sequences:		
Domain	N-terminus	C-terminus
c-myc tag	EQKLISEEDL	
CD8 hinge	VISNSVMYFS	SVKGTGLDFA
CD28 transmembrane and cytoplasmic	ALVVVAGVLF	PARDFAAYRP
CD3ζ cytoplasmic	LRAKFSRSAE	ALHMQTLAPR
Human CAR sequences:		
Domain	N-terminus	C-terminus
CD8 hinge	SALSNSIMYF	GGAVHTRGLD
CD28 transmembrane and cytoplasmic	FWVLVVVGGV	PPRDFAAYRS
CD3ζ cytoplasmic	RVKFSRSADA	ALHMQALPPR

### Generation of CAR lentiviruses

Human variants of scFv28z and DARPin28z were constructed in which the membrane proximal CD8 hinge region, the transmembrane and cytoplasmic regions of CD28, and the cytoplasmic region of CD3ζ of the murine CARs were replaced with the corresponding regions of their human counterparts (Table 1) (again, the cDNA sequence was synthesized at Genscript). Human scFv28z and DARPin28z CAR sequences were cloned into the lentiviral vector pCCL ΔNGFR (used for the generation of CAR –‘ve transduced human T cells) [29] (kindly provided by Dr. Megan Levings, University of British Columbia, Vancouver, BC). CARs were cloned downstream of the human EF1α promoter leaving ΔNGFR intact downstream of the minimal cytomegalovirus promoter in a bicistronic vector, pCCL. Third generation lentiviruses were made for each CAR construct; 8 × 10^6^ HEK 293 T cells in 15 cm tissue culture treated dishes (NUNC) (cultured in DMEM supplemented with 10 mM HEPES, 2 mM L-glutamine, 10 % FBS, and 0.1 mg/mL normocin (Invivogen, San Diego, CA)) were transfected with plasmids pRSV-Rev (6.25 μg), pMDLg-pRRE (12.5 μg), pMD2.G (9 μg), and CAR-encoding pCCL (30 μg) (kindly provided by Dr. Megan Levings, University of British Columbia, Vancouver, BC) using Lipofectamine 2000. After overnight incubation, media was replaced and supplemented with 1 mM sodium butyrate (Sigma-Aldrich). 30 h later, supernatants were harvested, filtered (0.45 μm), and concentrated (4 °C, 1 hr 40 min, 1.3 × 10^5^rcf). Viral stocks were resuspended in PBS and stored at −80 °C. Thawed virus aliquots were titrated by serial dilution and transduction of HEK 293 T cells to determine transduction units per milliliter.

### Transduction of murine T cells

Female BALB/c and C57BL/6 mice were purchased from Charles River Breeding Laboratory (Wilmington, MA). All of our investigations have been approved by the McMaster Animal Research Ethics Board. To generate murine T lymphoctes for retroviral transduction, 3 × 10^6^ freshly isolated splenocytes were cultured in 1 mL T cell media (RPMI supplemented with 10 % FBS, 2 mM L-glutamine, 10 mM HEPES, 0.5 mM sodium pyruvate, 0.1 mM non-essential amino acids, 55 μM β-mercaptoethanol, and 0.1 mg/mL normocin *or* 100U/mL penicillin + 100 μg/mL streptomycin) supplemented with 0.3 μg/mL α-CD3e (clone 145-2C11, Cat No. 553057, BD Pharmingen, San Diego, CA) and 400U/mL rhIL-2 (Cat No. 200–02, Peprotech, Rocky Hill, NJ). Twenty-four hours after activation, 600 μL of media were removed from T cell cultures and 100 μL of the concentrated retroviral supernatant was added, along with 2 μg/mL Lipofectamine 2000 and 1.6 μg/mL Polybrene (Sigma-Aldrich). Cultures were spun at 2000 rpm, 32 °C for 90 min, allowed to rest for 1–4 h, and were supplemented with 0.5 mL T cell media + 400U/mL rhIL-2. This process was repeated at 48 h after activation with the 72 h retroviral concentrates. Seventy-two hours after activation, retrovirally transduced T cell cultures were expanded into 30 mL of DC media + 400U/mL rhIL-2. Six to eight days after activation, resultant CAR-T cells were enumerated for use *in vitro*.

### Transduction of human T cells

Peripheral blood mononuclear cells from healthy donors were obtained using Ficoll-Paque-Plus (GE Healthcare, Baie d’Urfe, QC) separation. This research was approved by the McMaster Health Sciences Research Ethics Board that operates in compliance with the ICH Good Clinical Practice Guidelines, the Tri-Council Policy Statement: Ethical Conduct for Research Involving Humans, Division 5 Health Canada Food and Drug Regulations, and the Helsinki Declaration. All donors in this study provided informed written consent. 1 × 10^5^ cells were activated with anti-CD3/CD28 beads at a 1:1 ratio (Dynabeads, Cat No. 11131D, Life Technologies) in a 96-well round bottom plate (cultured in T cell media) with 100U/mL rhIL-2 and 10 ng/mL rhIL-7. Twenty-four hours after activation, T cells were transduced with lentivirus at an MOI of 1:1. CAR-T cell cultures were expanded into fresh media (T cell media supplemented with 100U/mL rhIL-2 and 10 ng/mL rhIL-7) as required for a period of 10–15 days prior to enumeration and use *in vitro*.

### Flow cytometry

Detection of CAR constructs on the surfaces of murine or human T lymphocytes was determined by indirect immunofluorescence with HER2Fc chimeric protein (Cat No. 1129-ER-050, R&D Systems, Minneapolis, MN) followed by a phycoerythrin (PE)-conjugated goat anti-human IgG (Cat No. 109-115-098, Jackson ImmunoResearch, West Grove, PA) *or* anti-myc-tag (clone 9B11, Cell Signaling Technology, Danvers, MA) followed by a PE-conjugated goat anti-mouse IgG (Cat No. 115-116-146, Jackson ImmunoResearch). Cell surface phenotyping of murine CAR-T cells was determined by direct staining with AlexaFluor(AF)700-conjugated anti-CD8a (clone 53–6.7, eBioscience Inc., San Diego, CA), PerCP-Cy5.5-conjugated anti-CD8a (clone 53–6.7, BD Pharmingen), PE-conjugated anti-CD8b (clone H35-17.2, BD Pharmingen), PE-conjugated anti-CD90.1 (Thy1.1, clone OX-7, BD Pharmingen), and/or fluorescein isothiocyanate (FITC)-conjugated anti-CD90.1 (clone HIS51, eBioscience Inc.). Cell surface phenotyping of human CAR-T cells was determined by indirect staining with HER2Fc chimeric protein (as for murine T cells) and direct staining with PE-CF594-conjugated anti-CD271 (NGFR, clone C40-1457, BD Biosciences), and AF700-conjugated anti-CD8a (clone OKT8, eBioscience Inc.). All flow cytometry was conducted on a BD FACSCanto or BD LSRII cytometer (BD Bioscience) and analyzed using FlowJo vX.0.7 software (FlowJo, LLC, Ashland, OR, USA).

### Functional analysis of CAR-T cells following stimulation with recombinant protein

10^6^ murine or human CAR-T cells were stimulated in round bottom tissue culture treated 96-well plates coated with 200 ng HER2Fc chimeric protein (Cat No. 1129-ER-050, R&D Systems) or 200 ng KDRFc chimeric protein (Cat No. 443-KD, R&D Systems) for 4 h at 37 °C. Protein transport was inhibited according to the BD Golgi Plug protocol (Cat No. 555029 BD Biosciences, San Diego, CA). Production of activation cytokines was determined by flow cytometry. Cells were stained for surface phenotype markers as above. To permit intracellular cytokine staining (ICS), CAR-T cells were fixed and permeabilized according to BD Cytofix/Cytoperm Fixation/Permeabilization Kit protocol (Cat No. 554714, BD Biosciences). ICS of murine CAR-T cells was conducted by direct staining with allophycocyanin (APC)-conjugated anti-IFN-γ (clone XMG1.2, BD Pharmingen) and PE-cyanine(Cy)7-conjugated anti-TNF (clone MP6-XT22, BD Pharmingen). ICS of human CAR-T cells was conducted by direct staining with APC-conjugated anti-IFN-γ (clone B27, BD Pharmingen), FITC-conjugated anti-TNF (clone MAb11, BD Pharmingen), PE-conjugated anti-IL-2 (clone MQ1-17H12, BD Biosciences), and FITC-conjugated anti-CD107a (clone H4A3, BD Pharmingen). Analysis and presentation of distributions was performed using SPICE version 5.1, downloaded from [30]. Comparison of distributions was performed using a Student’s T test and a partial permutation test as described [31], with a threshold of 0.09.

### In vitro cytotoxicity assay

Adherent tumor cell lines were plated at 1.25 × 10^4^ cells/well (D2F2, D2F2/E2, HCC1954, or LOX-IMVI) or 1.25 × 10^4^ (human CAR-T cell cytotoxicity assay) *or* 2.5 × 10^4^ cells/well (murine CAR-T cell cytotoxicity assay) (SKBR3) overnight in 96-well flat bottom tissue culture treated plates. Transformed murine or human T cell cultures were added to wells of tumor cells at various E:T ratios (from 0.1:1 to 6:1) and co-incubated together at 37 °C for 6 h. Murine T cells were added based on effectors defined as cells from day 6 to 8 transduced murine T cell cultures. Human T cells were added based on effectors defined as CAR-positive cells from day 15 transduced human T cell cultures. Wells were washed 3× with warmed PBS to remove any non-adherent cells. 100 μL of a 10 % solution of AlamarBlue cell viability reagent (Life Technologies) in T cell media was added and wells were incubated at 37 °C overnight. Colour change, indicative of live cells, was measured by fluorescence (excitation 530 nm, emission 595 nm) on a Safire plate reader (Tecan, Maennendorf, Switzerland). Tumor cell viability was calculated as the loss of fluorescence in experimental wells compared to untreated target cells. Each condition was tested in triplicate.

### Statistical analysis

Student’s *t* tests, two-tailed, type two, were used to compare data between two groups. Results were prepared using Microsoft Excel 2010. Significant differences were defined as: * = *p* < 0.05, ** = *p* < 0.01, *** = *p* < 0.001; NS = not significant.

## Abbreviations

AR: ankyrin repeat
CAR: chimeric antigen receptor
CAR-T cell: chimeric antigen receptor transduced T cell
DARPin: designed ankyrin repeat protein
E: effector
scFv: single-chain variable fragment
SD: standard deviation
SE: standard error
T: target
